# Reference centiles based on year-to-year changes for a longitudinal evaluation of motor performance in children and adolescents

**DOI:** 10.1371/journal.pone.0262163

**Published:** 2022-01-07

**Authors:** Sven Wessela, Christof Meigen, Tanja Poulain, Carolin Sobek, Mandy Vogel, Siegfried Möller, Wieland Kiess

**Affiliations:** 1 LIFE Leipzig Research Center for Civilization Diseases, Leipzig University, Leipzig, Germany; 2 Department of Women and Child Health, Hospital for Children and Adolescents and Center for Pediatric Research (CPL), Leipzig University, Leipzig, Germany; Illawarra Shoalhaven Local Health District, AUSTRALIA

## Abstract

**Objectives:**

The aim was to evaluate the longitudinal course of motor skills development in children with a view to improve the understanding of intra-individual variance. Previous publications have been based on cross-sectional data or analyzed longitudinal studies in a cross-sectional manner.

**Design:**

Longitudinal. Year-to-year change.

**Methods:**

The present study is based on motor function data collected in the LIFE Child study (Germany). The participants (6 to 17 years) completed parts of the motor tests according to the standard of the German Motor Performance Test 6–18 (DMT). For a total of 1653 participants, 4616 motor tests with an annual interval in the period 2011 to 2019 were included in the evaluation.

**Results:**

We were able to produce gender and age-specific change centiles for the test items *standing long jump*, *jumping sideways*, *push-ups*, *stand and reach* and *balancing walking backwards*. Each set of centiles revealed a range of variability in motor development in children and adolescents, with distinct progressive patterns in the different test items and different genders. The supplied tables offer an indication of expected year-to-year change for each test item depending on age and gender. Depending on the test item and the mean age, a deterioration in test results after a one-year interval was observed, despite cross-sectional centiles showing an upward trend.

**Conclusion:**

We present a user-friendly tool as a way to assess individual dynamic changes in motor development of children and adolescents. In combination with the well-known cross-sectional centiles as baseline, this can be helpful for the scientific evaluation of motor skills tests and can also be used in school settings.

## Introduction

Motor competence is a growing area of research, particularly in children and adolescents [1]. In addition to associations with anthropometric data, previous studies also investigated correlations with physical activity, assessed via questionnaires [2, 3] or accelerometry [4–6], cardiorespiratory fitness [7, 8] and motor skills [9]. Several studies assessed associations with different aspects [10, 11].

For the most part, previous motor skill studies have used a cross-sectional or cohort design [12–14]. In several of the published studies that have used longitudinal evaluation methods, the test subjects were grouped by age and gender, and cross-sectional analyses were performed. This has indicated an age-related increase in the performance of the respective test item [15].

A number of previous studies focused on the physical development of adolescents over many years or decades [15–17]. The results of longitudinal studies, among other aspects, underline the problem of low stability and confirm that physical activity is a fluctuating variable [3].

There are already studies that refer to longitudinal measurement results of motor skills in their correlation analysis. However, an individual estimation of the development of motor skills cannot be read out for the respective test subjects [18, 19].

In addition to the known cross-sectional centiles [15], which correspond to a baseline, we believe that individual longitudinal observations of motor function data, which correspond to the slope in the development function, should be integrated into the assessment of the physiological development of children and adolescents. While cross-sectional centiles can be used as a visual guideline to assess whether children develop “along their centiles”, they do not allow to quantify the extent of any deviation from normal development patterns.

A simple approach to evaluating longitudinal data would be to document and assess change in measured values in a defined time interval (Δ = t2-t1). Such an assessment could utilize change centiles, in the same way in which they are already used to assess child development, e.g., centile curves for growth rate [20].

To our knowledge, there is no comparable work in the literature that allows the user to assess changes in individual motor performance in children and adolescents to disciplines of the German Motor Performance Test 6–18 (DMT). This may be of interest to both scientists and sports education professionals.

## Design and methods

### Participants of the LIFE Child study

The present study is based on motor function data collected as part of the LIFE Child study. The LIFE Child study is a longitudinal study conducted at the Research Centre for Civilization Diseases in Leipzig (Germany). It aims to investigate development in children and adolescents with a particular focus on the development of lifestyle diseases. The comprehensive study program includes various medical, psychological and socio-demographic assessments and the collection of biological samples. Standardized data collection, process control and data analysis are ensured by a professional team of physicians, certified study assistants, quality managers, scientists and statisticians [21–23]. The study cohort consists of healthy children and adolescents. The ethnicity of the subjects was not recorded in the LIFE Child study approach.

In accordance with the Declaration of Helsinki [24]. this study was designed and approved by the Ethics Committee of the Medical Faculty of the University of Leipzig (Chair: Prof. Ortrun Riha, Reg. No. 264/10-ek, date of last approval: 3 December 2020). The ethics vote is available in written form.

### Measurements

The measurement was conducted according to the DMT standard (German Motor Performance Test 6–18 years). The DMT is a well-established and validated method of measuring physical motor performance in children and adolescents [25–28]. In its original form, the DMT 6–18 consists of 8 test items: standing long jump, jumping sideways, push-ups, stand and reach, balancing walking backwards, sit-ups, 20-meter sprint, 6-minute run. Only the first five of these tests were conducted as part of the LIFE Child study program. This limitation of the scope of the motor examination was done for reasons of feasibility and resources. According to Oberger et al. [25], the average test-retest reliability was given as a coefficient of 0.86, indicating good test reliability.

### Modelling

#### Longitudinal approach

The evaluation presented here is based on motor data from children and adolescents who performed motor tests at approximately one-year intervals and contains results from 9275 separate measurements. To ensure the highest data quality and to verify the influence of motivation on the study results, only those measurement results were included in the evaluation for which documentation of good motivation was available by means of a quality management questionnaire. In order to represent the dynamic development of the participants, motor data of each individual subject were combined as a pair (*Δx = x*_*t2*_*-x*_*t1*_).

**Fig 1:**
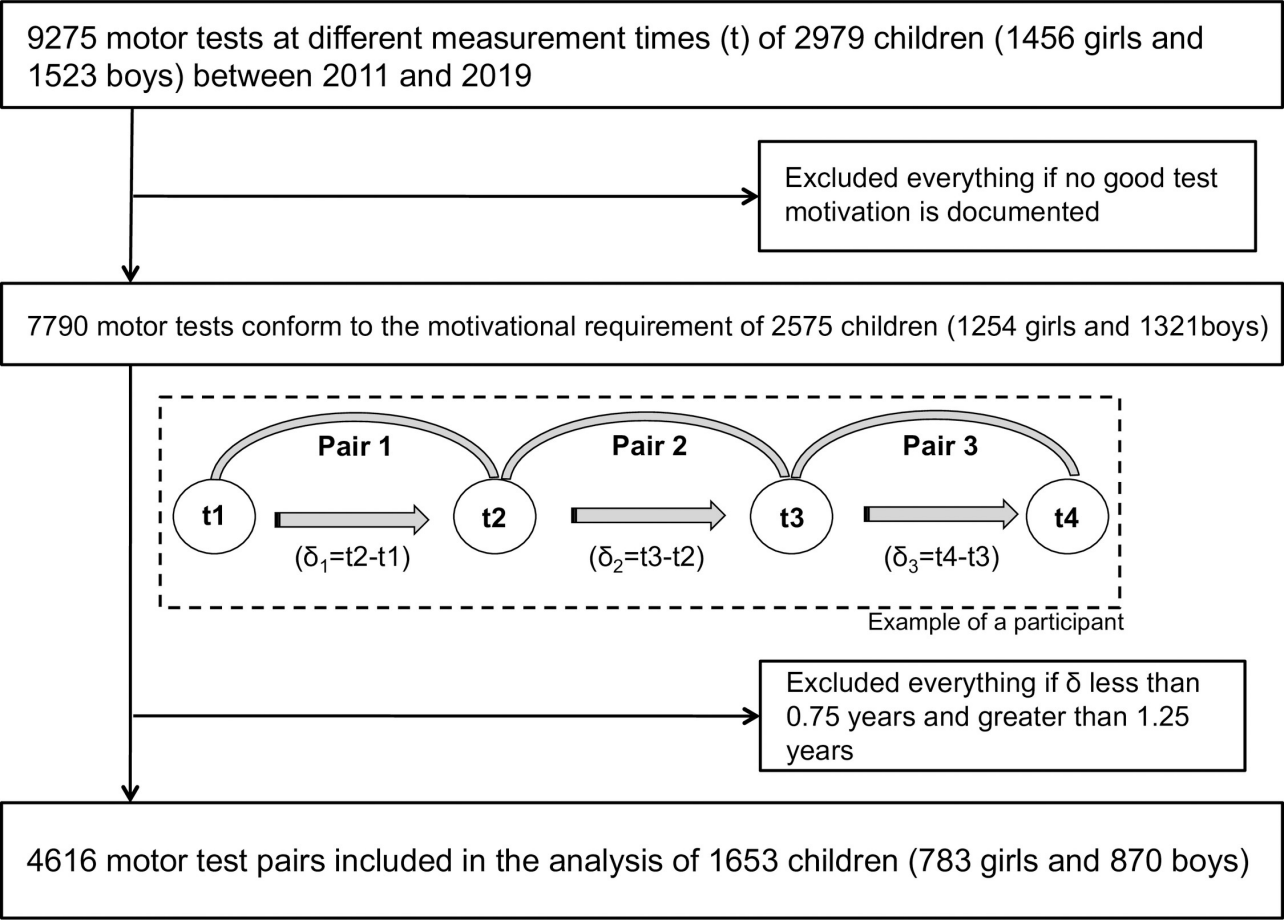
Flowchart to visualise final approach.

If the tests were at least 0.75 years apart and no more than 1.25 years apart, these were included in the analysis. Attached is a flowchart (Fig 1) based on the example of jumping sideways, which describes the subjects and measurements entered. The cross-sectional analysis including the respective mean values can be found in Möller et al. [23].

#### Normalisation to annual interval

As is common practice, the rescreening appointments took place at annual intervals. Since the age, or age differences of participants would have a corresponding influence on the test result, it was determined that only pairs of measurements were included that could be conducted at an interval of at least 0.75 years and at most 1.25 years. Measurement intervals shorter or longer were not taken into account. In order to normalise the respective measurement intervals to 1 year, the values were divided by the time interval (*Δx*_*n*_ *= Δx/Δt*).

#### Weighting of several pairs of a single participant

If it was possible to include more than one pair of test values for a single subject in the analyses (occurred for 1162 participants), these were weighted proportionally, i.e., if two pairs were included for a particular participant, each pair was given a weighting of 50%. If there were more pairs, the weighting was reduced proportionally.

### Statistical analysis

R, the free programming language for statistical calculations and graphics (version 3.5.1 for OS X; R Foundation for Statistical Computing, Vienna, Austria [2018]) was used for data preparation and analysis. To calculate the change centiles, the Skew Power exponential type 2—SEP2 (GAMLSS version 5.1–4) [29] was applied. For this distribution, parameters for the mean (μ), standard deviation (σ) and skewness (ν) are modeled depending on age. For the model, the following parameters were selected: μ: ~pb (mean age, 3), log(σ): ~pb (mean age, 3), ν: ~mean age. As usual, the smoothing parameters were chosen after inspecting the distributions of all motoric tests as a compromise between smooth centile curves and a good fit of the distribution. To make the centile curves–which all reflect motoric development–comparable, it was decided to use the same smoothing parameters for all motoric tests. Note, “v” is supposed to mean the Greek letter “nu”.

## Results

### Participant characteristics

A total of 1653 children (783 girls, 870 boys; age range = 5.97 to 17.97 years) met the criteria for inclusion. Table 1 shows the distribution of age, sex, SES and BMI among the participants.

**Table 1:** Descriptive statistics to characterise the participants included in the model.

	Female (n = 783)										
Mean age (y)	6	7	8	9	10	11	12	13	14	15	16
Test pairs (n)	135	223	213	231	221	214	238	237	208	189	139
sex %	50.9	47.5	42.9	44.2	44.7	46.6	46.9	46.8	48.1	48.8	49.1
BMI	15.3	15.8	16.4	16.89	17.67	18.43	19.5	20.4	21.3	21.9	22.1
	±1.70	±2.35	±2.76	±3.04	±3.43	±3.86	±4.06	±4.14	±4.17	±4.53	±4.01
BMI (%):											
obese	2.2	5.4	6.1	6.5	8.6	8.4	10.5	10.1	11.1	9.0	10.1
overweight	3.7	3.6	4.2	4.8	5.4	5.1	5.9	9.7	8.2	7.9	9.4
normalweight	83.7	83.9	82.6	81.0	77.4	76.2	73.9	73.4	74.5	77.8	74.8
underweight	10.4	7.2	7.0	7.4	8.1	9.8	9.7	6.8	6.2	5.3	5.8
SES (%):											
high	35.0	30.1	29.1	28.2	28.1	30.4	25.7	25.0	23.4	23.6	23.1
middle	57.3	59.6	60.3	61.2	58.9	56.4	62.1	63.5	63.5	60.8	59.6
low	7.7	10.4	10.6	10.6	13.0	13.3	12.1	11.5	13.2	15.5	17.3
	Male (n = 870)										
Mean age (y)	6	7	8	9	10	11	12	13	14	15	16
Test pairs (n)	130	246	284	292	273	245	269	269	224	198	144
sex %	49.1	52.5	57.1	55.8	55.3	53.4	53.1	53.2	51.9	51.2	50.9
BMI	15.5	15.61	16.04	16.91	17.3	17.91	19.5	20.1	20.5	21.2	21.3
	±1.46	±1.52	±2.22	±3.05	±3.07	±3.28	±3.91	±4.04	±3.95	±4.22	±3.74
BMI (%):											
obese	3.8	2.8	4.6	7.9	7.0	6.1	10.4	10.0	8.9	8.6	4.9
overweight	1.5	2.8	2.8	4.1	3.7	4.9	8.6	11.2	10.3	9.1	8.3
normalweight	87.7	87.4	85.6	77.7	80.2	78.4	72.5	68.0	72.3	73.7	79.2
underweight	6.9	6.5	6.7	9.9	9.2	10.2	8.6	10.8	8.5	8.6	7.6
SES (%):											
high	31.6	32.7	34.0	31.5	31.8	30.4	27.0	23.1	25.0	25.3	23.3
middle	66.7	61.1	58.4	58.8	58.1	58.5	61.1	66.5	67.2	67.1	63.1
low	1.8	6.2	7.6	9.7	10.2	11.1	11.9	10.4	7.8	7.5	13.6

BMI: body mass index, was calculated using weight (kg) and height (m), in kg/m^2^, Classification according to Kromeyer-Hauschild et al. [30]; SES: socio economic status, Classification according to Lampert et al. [31].

Furthermore, under Supporting information (see S1 Table), a presentation of participants split by test item, gender and frequency of inclusion in the model can be found.

### Change centiles plots

**Fig 2:**
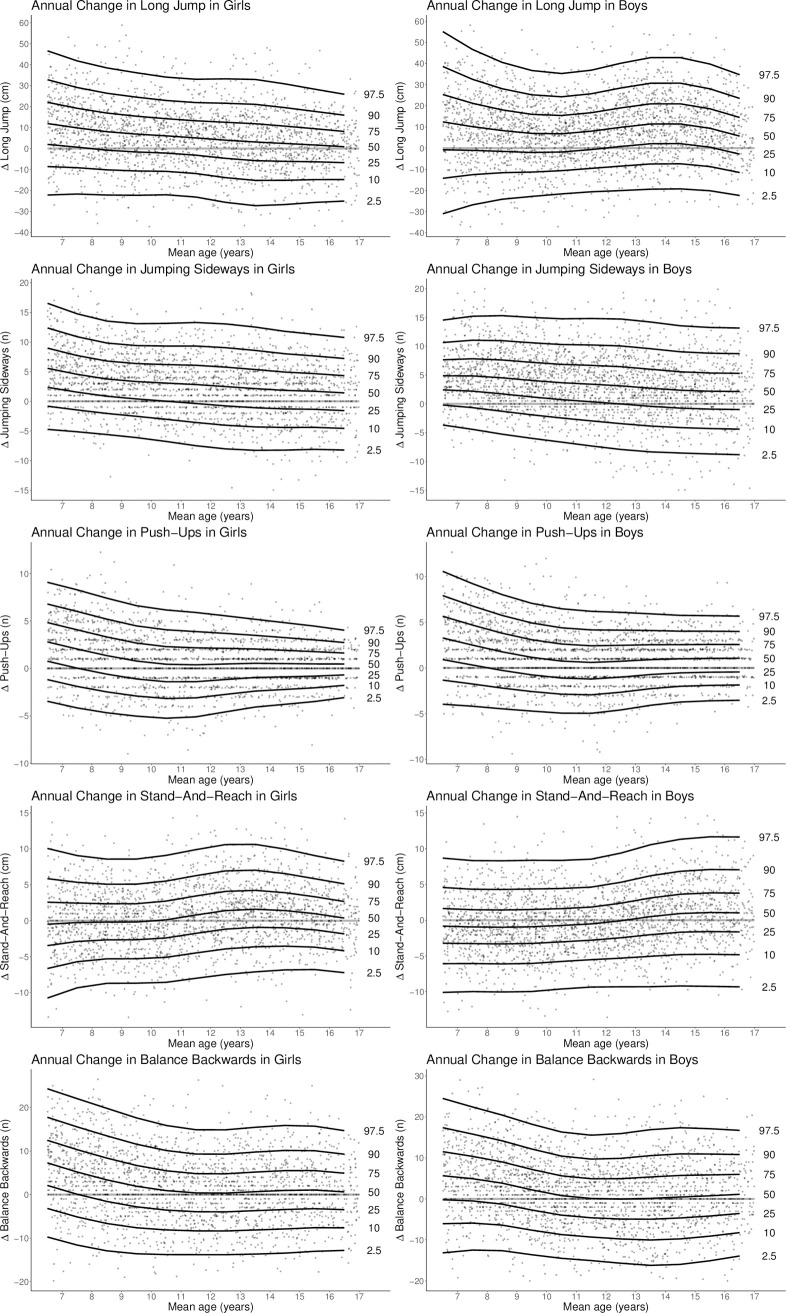
Change centile plot. The diagrams are scaled dependent on gender and test items. The x-axis represents the mean age of a measurement pair. The plots show on the y-axis the changes in measured values (Δ = t2-t1) in an annual interval for the respective test persons and test items. With reference to the point distribution, the percentile curves illustrated are obtained. Gradual gender-specific and item-specific differences in the dynamics of development can be observed.

Changes in motor performance in our sample of children and adolescents are illustrated in Fig 2, with the changes in results between the first and second tests in each pair (Δ = t2-t1) indicated on the y-axis in each centile diagram. The diagrams appear in the following order: standing long jump, jumping sideways, push-ups, stand and reach and balancing walking backwards. Standard centile divisions of 2.5^th^, 10^th^, 25^th^, 50^th^, 75^th^, 90^th^ and 97.5^th^ have been used. The null level of the test items has been embedded for easier orientation. The x-axis represents the mean age of the participant for the relative measurement pair.

Even an initial, summary examination of the diagrams, the different genders and different motor tests present clear variations in terms of the pattern of progressive variation in the centiles of change as the age of the participants increases. We can attempt to characterize these patterns in the development of the centiles in visual terms: more-or-less straight, parallel centile lines with a gradual shift up or down (standing long jump and jumping sideways in girls); percentile lines gradually converging [towards a point] as age increases (push ups in both genders); a wave or buckle in the centile lines (standing long jump in boys as well as stand and reach in girls). The approximately straight, parallel centiles in standing long jump or jumping sideways in girls indicate that the level of variation for these test items neither increases nor decreases in relation to age.

The results for the push-ups test are characterized by a convergence in the centile lines with increasing age, indicating a reduction in intra-individual variability proportional to age. However, the 50^th^ centile remains above the null level, which means that, on average, there has been an annual increase in the number of push-ups counted. Compared to females, male participants show a higher level of individual improvement.

The opposite pattern, a “fanning out” or diverging trend (jumping sideways as well as stand and reach in boys) means that the range of variability increases with age, with larger positive or negative changes between the earlier measurement in a given pairing and the later measured value. In part, this may be due to an increase in absolute values, which allow for larger changes.

Waves or buckles in the centile lines seem to occur mostly during puberty, suggesting a period of changed variability for certain scores. It should be noted that a drifting apart of the centile curves indicates an increase in variability. For the standing long jump, there is a difference between genders. Among males, there is a positive wave with a peak in the 14^th^ year of age, while females show no similar effect during puberty. In comparison, there is a positive wave in both genders in the stand and reach test, with the wave peaking at the age of 13 in the female participants, two years earlier than among males.

### Change centiles table

Correspondingly, Table 2 shows the numerical values for change centiles for standing long jump, jumping sideways, push-ups, stand and reach and balancing walking backwards, separated by gender, again using the standard centile divisions of 2.5^th^, 10^th^, 25^th^, 50^th^, 75^th^ 90^th^ and 97.5^th^. These should only be used in relation to changes in results over time intervals between 0.75 and 1.25 years, consistent with how the centiles themselves were calculated.

**Table 2:** Change centiles. Change centile tables scaled by gender, average age and test items, the longitudinal development between two measurement points within a year can be evaluated.

	Mean age (y)	Female									Mean age (y)	Male							
Centiles		2.5^th^	10^th^	25^th^	50^th^	75^th^	90^th^	97.5^th^	(n)	Centiles		2.5^th^	10^th^	25^th^	50^th^	75^th^	90^th^	97.5^th^	(n)
Standing long jump (cm)	6	-22.2	-8.6	2.0	11.9	22.1	32.9	46.6	119	Standing long jump (cm)	6	-31.0	-14.2	-0.8	12.4	25.3	38.6	55.1	106
	7	-21.7	-9.2	0.6	9.9	19.2	29.1	41.8	216		7	-26.8	-12.5	-1.1	10.2	21.2	32.5	46.6	233
	8	-22.2	-10.2	-0.8	8.0	16.9	26.4	38.5	204		8	-24.1	-11.6	-1.5	8.3	18.1	28.1	40.5	271
	9	-22.3	-10.7	-1.7	6.9	15.4	24.5	36.2	226		9	-22.8	-11.3	-2.1	7.0	16.0	25.2	36.7	275
	10	-22.1	-10.9	-2.2	6.1	14.2	22.9	34.1	212		10	-21.6	-10.6	-1.8	6.8	15.4	24.3	35.3	261
	11	-23.2	-11.9	-3.2	5.1	13.2	21.9	33.1	204		11	-20.6	-9.6	-0.7	8.0	16.8	25.7	36.8	235
	12	-25.7	-13.9	-4.7	4.0	12.5	21.6	33.3	223		12	-20.0	-8.5	0.8	9.9	19.1	28.5	40.1	256
	13	-27.3	-15.2	-5.8	3.1	11.8	21.1	33.0	214		13	-19.4	-7.5	2.1	11.4	21.0	30.7	42.8	260
	14	-26.7	-15.2	-6.1	2.4	10.7	19.5	30.9	196		14	-19.2	-7.4	2.1	11.4	21.0	30.7	42.8	218
	15	-25.7	-14.9	-6.3	1.7	9.5	17.7	28.3	178		15	-20.1	-8.7	0.5	9.4	18.6	28.0	39.8	192
	16	-25.1	-14.8	-6.8	0.8	8.1	15.9	25.9	133		16	-22.4	-11.5	-2.8	5.7	14.5	23.5	34.7	141
Centiles		2.5^th^	10^th^	25^th^	50^th^	75^th^	90^th^	97.5^th^	(n)	Centiles		2.5^th^	10^th^	25^th^	50^th^	75^th^	90^th^	97.5^th^	(n)
Jumping sideways (n)	6	-4.7	-0.8	2.4	5.6	9.0	12.4	16.5	115	Jumping sideways (n)	6	-3.7	-0.2	2.4	4.9	7.7	10.7	14.5	104
	7	-5.1	-1.5	1.5	4.6	7.7	10.9	14.8	210		7	-4.4	-0.7	2.2	4.9	7.8	11.1	15.2	229
	8	-5.6	-2.1	0.9	3.8	6.8	9.8	13.5	207		8	-5.3	-1.3	1.7	4.5	7.6	11.0	15.3	266
	9	-6.1	-2.5	0.4	3.3	6.4	9.4	13.1	229		9	-6.0	-1.9	1.2	4.1	7.2	10.6	15.0	278
	10	-6.8	-3.0	0.0	3.1	6.2	9.3	13.2	216		10	-6.7	-2.5	0.7	3.7	6.9	10.3	14.8	255
	11	-7.5	-3.6	-0.4	2.8	6.1	9.3	13.3	211		11	-7.3	-3.0	0.4	3.4	6.7	10.2	14.8	233
	12	-8.0	-4.0	-0.8	2.5	5.8	9.1	13.1	234		12	-7.9	-3.4	0.0	3.1	6.5	10.1	14.7	258
	13	-8.3	-4.3	-1.1	2.1	5.4	8.6	12.5	225		13	-8.3	-3.9	-0.4	2.7	6.0	9.6	14.2	259
	14	-8.2	-4.4	-1.3	1.8	4.9	8.0	11.8	204		14	-8.5	-4.1	-0.7	2.4	5.6	9.1	13.6	215
	15	-8.1	-4.4	-1.4	1.7	4.7	7.7	11.3	183		15	-8.7	-4.3	-0.9	2.2	5.4	8.8	13.3	193
	16	-8.2	-4.6	-1.6	1.4	4.3	7.2	10.8	133		16	-8.8	-4.4	-1.0	2.2	5.3	8.7	13.2	142
Centiles		2.5^th^	10^th^	25^th^	50^th^	75^th^	90^th^	97.5^th^	(n)	Centiles		2.5^th^	10^th^	25^th^	50^th^	75^th^	90^th^	97.5^th^	(n)
Push-ups (n)	6	-3.5	-1.2	0.8	2.8	4.8	6.8	9.1	103	Push-ups (n)	6	-4.0	-1.3	0.9	3.3	5.6	7.9	10.5	89
	7	-4.2	-1.9	0.0	2.0	4.1	6.0	8.3	202		7	-4.2	-1.8	0.3	2.5	4.7	6.8	9.2	210
	8	-4.7	-2.5	-0.6	1.4	3.3	5.2	7.4	201		8	-4.5	-2.2	-0.3	1.8	3.8	5.7	8	263
	9	-5.0	-2.9	-1.1	0.8	2.7	4.5	6.6	223		9	-4.7	-2.6	-0.8	1.2	3.1	4.9	7.1	272
	10	-5.3	-3.2	-1.4	0.4	2.3	4.1	6.2	207		10	-4.9	-2.9	-1.1	0.8	2.6	4.4	6.5	260
	11	-5.1	-3.1	-1.4	0.4	2.2	3.9	5.9	198		11	-5.0	-2.9	-1.2	0.6	2.4	4.2	6.2	236
	12	-4.6	-2.7	-1.2	0.5	2.1	3.7	5.5	216		12	-4.6	-2.7	-1.0	0.7	2.5	4.1	6.1	253
	13	-4.1	-2.4	-1.0	0.5	2.0	3.5	5.2	216		13	-4.1	-2.3	-0.7	0.9	2.5	4.1	5.9	254
	14	-3.8	-2.2	-0.9	0.5	1.9	3.2	4.8	197		14	-3.7	-2.0	-0.5	1.0	2.5	4.0	5.7	214
	15	-3.5	-2.1	-0.8	0.5	1.8	3.0	4.5	172		15	-3.6	-1.9	-0.5	1.1	2.6	4.0	5.7	192
	16	-3.1	-1.8	-0.7	0.5	1.6	2.7	4.0	124		16	-3.5	-1.9	-0.4	1.1	2.6	4.0	5.7	139
Centiles		2.5^th^	10^th^	25^th^	50^th^	75^th^	90^th^	97.5^th^	(n)	Centiles		2.5^th^	10^th^	25^th^	50^th^	75^th^	90^th^	97.5^th^	(n)
Stand and reach (cm)	6	-10.7	-6.6	-3.5	-0.5	2.6	5.9	10.1	115	Stand and reach (cm)	6	-10.1	-6.1	-3.2	-0.8	1.6	4.6	8.7	100
	7	-9.3	-5.7	-2.9	-0.3	2.5	5.4	9.1	214		7	-10.0	-6.1	-3.3	-0.9	1.5	4.3	8.3	228
	8	-8.7	-5.3	-2.7	-0.2	2.4	5.1	8.6	208		8	-10.1	-6.1	-3.3	-1.0	1.4	4.3	8.3	276
	9	-8.7	-5.3	-2.7	-0.2	2.4	5.1	8.6	231		9	-10.0	-6.1	-3.3	-0.9	1.5	4.4	8.4	276
	10	-8.6	-5.1	-2.4	0.1	2.7	5.5	9.1	217		10	-9.6	-5.8	-3.0	-0.7	1.6	4.4	8.4	250
	11	-8.1	-4.5	-1.8	0.8	3.4	6.2	9.8	204		11	-9.4	-5.5	-2.8	-0.5	1.8	4.6	8.5	226
	12	-7.5	-4.0	-1.2	1.4	4.1	6.9	10.5	225		12	-9.3	-5.3	-2.5	-0.1	2.4	5.3	9.4	247
	13	-7.2	-3.7	-0.9	1.6	4.2	7.0	10.6	230		13	-9.3	-5.1	-2.0	0.5	3.1	6.2	10.6	248
	14	-6.9	-3.6	-1.0	1.4	3.9	6.5	9.9	201		14	-9.2	-4.8	-1.7	0.9	3.6	6.8	11.3	209
	15	-6.9	-3.7	-1.3	1.0	3.4	5.8	9.0	181		15	-9.3	-4.8	-1.6	1.1	3.8	7.1	11.7	190
	16	-7.3	-4.2	-1.8	0.4	2.7	5.1	8.2	136		16	-9.3	-4.8	-1.6	1.0	3.8	7.1	11.6	142
Centiles		2.5^th^	10^th^	25^th^	50^th^	75^th^	90^th^	97.5^th^	(n)	Centiles		2.5^th^	10^th^	25^th^	50^th^	75^th^	90^th^	97.5^th^	(n)
Balancing walking backwards (n)	6	-9.8	-3.2	2.1	7.3	12.5	17.8	24.3	115	Balancing walking backwards (n)	6	-13.2	-6.1	-0.2	5.7	11.5	17.4	24.5	99
	7	-11.6	-5.1	0.1	5.2	10.3	15.6	22.1	210		7	-12.5	-5.9	-0.5	4.9	10.4	15.8	22.4	210
	8	-12.9	-6.6	-1.6	3.4	8.4	13.5	19.8	205		8	-12.6	-6.4	-1.3	3.9	9.0	14.2	20.5	256
	9	-13.6	-7.6	-2.8	1.9	6.7	11.6	17.6	228		9	-13.6	-7.6	-2.7	2.2	7.2	12.2	18.2	273
	10	-13.8	-8.1	-3.6	0.9	5.5	10.1	15.9	215		10	-14.5	-8.7	-4.0	0.8	5.6	10.5	16.3	256
	11	-13.8	-8.3	-3.9	0.4	4.8	9.3	14.9	209		11	-15.1	-9.3	-4.6	0.1	4.9	9.7	15.6	231
	12	-13.8	-8.4	-4.0	0.3	4.8	9.3	14.9	234		12	-15.7	-9.8	-4.9	-0.1	4.9	9.9	15.9	253
	13	-13.7	-8.1	-3.7	0.7	5.2	9.8	15.5	228		13	-16.2	-10	-5.0	0.1	5.4	10.6	16.9	257
	14	-13.5	-7.9	-3.4	1.0	5.5	10.2	15.9	202		14	-16.0	-9.8	-4.7	0.5	5.7	11.0	17.4	210
	15	-13.1	-7.6	-3.2	1.0	5.5	10.1	15.7	186		15	-15.1	-9.1	-4.2	0.8	5.9	10.9	17.1	187
	16	-12.8	-7.7	-3.5	0.6	4.9	9.3	14.7	136		16	-13.9	-8.2	-3.6	1.1	6.0	10.8	16.7	140

Additionally, the last column shows the number of test participants that constitute the basis for the evaluation. For each of the age groups from 7 to 13 years, data was available for 200 or more participants; for ages 6 and 14–16 years, the centiles are based on data from fewer subjects.

The tables can be used as a tool to evaluate changes in the performance of a test subject in a defined age group. By providing reference values for the change in measured test performance over a one-year time period broken down by centile (column) and mean age (row), they offer a metric for evaluating longitudinal development between two measuring points within one year.

## Discussion

To respond to the question how to assess changes in motor test scores of children and adolescents in an annual interval, we generated centile curves for the change in performance between two identical tests settings (in the context of the LIFE Children’s Study). The implementation of centiles for cross-sectional motor skill measurement is not new. For example, Goble et al. [32] described percentiles separated by gender for a balance test. As far as concerns data from the DMT 6–18 motor performance test, this is the first presentation of change centiles in a longitudinal model.

### Centiles curves interpretation

Furthermore, the centile curves provide a clear indication of the different gender-dependent effects of puberty on the test items. For example, for the standing long jump test, a decrease in performance in the 15^th^ year is less likely than in other age groups in males, but not in females. For stand and reach, there is a peak in both genders, although it occurs about 2 years earlier in girls than in boys. This wave can also be seen in the centiles values (Table 2). Here, the 50^th^ centile in stand and reach in girls between the ages of 10 and 16 initially shows an upward trend, followed by a downward trend. However, there is also a test item (push-ups) where no gender-specific difference in variability is obvious. Longitudinal differences in standing long jump performance during puberty is also described by Silva et al. [33].

### Study population

A comparable study approach [15] shows a similar result for SES. In terms of BMI, the LIFE Child cohort has a higher proportion of obese children and adolescents, but fewer overweight children and adolescents.

### Modelling

The choice of one year as an interval between measurements has already been used in other motor performance studies [10, 34] and corresponds to the cycle of grades in a school setting.

The question of weighting the measured values, to account for drop-outs or multiple measurements of a single participant, was a point in the analysis that should not be neglected. A weighting procedure is important to reduce longitudinal bias in health oriented epidemiological studies [35].

### DMT assessment

The test instruction and evaluation of the DMT remains a factor to be discussed although validity and reliability for the DMT have already been extensively analysed [25–27]. The various test results are included in the evaluations in different ways. While an average value is calculated from the two measurements for the jumping sideways test, the best value remains for the standing long jump and the stand and reach test. For the push-ups and balancing walking backwards the complete performance is included in the evaluation. It remains to be investigated whether or how the relationship between day-to-day and year-to-year variability affects the modelling of physiological development in children and adolescents.

For the test administration in the LIFE Child study, it can be said that there were documented quality points for the bulk of the test assessments, such as the children’s motivation. These suggest a high level of motivation for the individual tests.

### Cross-sectional vs. longitudinal approach

So far, the results of cross-sectional studies have suggested that physical performance changes monotonically from year to year. This analysis now shows that the changes are variable and can also be negative. Cross-sectional studies have not yet provided reliable information on longitudinal trends. For example, interpretation of e.g. the decline from 50^th^ to 10^th^ percentile would not be totally meaningful using the cross-sectional approach. The change centiles differ from the known courses of the cross-sectional curves on the one side in their shape and on the other side in their variability. A broader scattering of values is evident here, since this is not averaged in the respective age group as in cross-sectional studies. For the assessment of physiological development, the change centiles for individual assessment can be combined with the known cross-sectional curves as a baseline.

### Rate of deterioration in annual comparison

As a main point, it has been shown that performance deterioration within one year is not unusual. Depending on the test, a deterioration in year-to-year development could be found in 10% to 50% of the subjects. For example, 25% of 12 year old boys achieve a worse result in sideways jumping than in the previous year. This is particularly noteworthy as the known cross-sectional percentiles indicate a increase in motor performance with age. The average performance of the children, corresponding to the 50^th^ centile, shows the group trend towards yearly improvement.

### Variability

The authors were surprised by the variability of the individual results in a year-to-year comparison. In terms of the findings from this paper, there was more variability than a naive interpretation of the available cross-sectional data would suggest. The range of variability of the children is the basis for the range of the centiles. When generating the change centiles, the pronounced variability of the test results is immediately apparent, with the values in the upper and lower centiles (2.5^th^ and 97.5^th^) indicating a high degree of variability in a single individual’s performance. The intra-individual variability has implications for how such test results might be interpreted. General health aspects like body mass index (BMI), daily condition, random effects or the motivation of the children and adolescents in question may need to be taken into account, while the test set-up should also be reviewed when assessing highly variable values.

### Science application

The aim of the article is to create a ready-to-use instrument for assessing the motor development of children and adolescents.

A variety of applications can be imagined for the proposed change centiles, especially in the table format. In addition to the evaluation of an individual’s performance in longitudinal settings, this tool can also be used for the general quality control of motor tests and can be integrated into the evaluation process as a plausibility check, where it can help provide a suitable estimation of the validity of a measured value with respect to previous measurements. The individual items are also part of many other motor test formats. The single items are not exclusive to the DMT. From the authors’ perspective, it is certainly interesting to see the extent of annual variability. Comparing one’s own measurements with the change centiles can be used as a basis for deciding when a result is alarming and a further history, paediatric examination or re-testing should be recommended. For sport scientists who use other or similar tests and have comparable data, this article can be a template for creating a similar instrument for longitudinal assessment.

### School application

Similar to the LIFE Child cohort, a heterogeneous subpopulation is also found in the school setting. It is not uncommon for children and adolescents in an age group to differ by more or less than one year, to be of different heights or weights, and for their physical activity to vary. It is also not uncommon that these covariates are not included in the assessment of athletic performance, leading to subjectively unfair results. Assessing pupils’ performance development can be one way to make this grading somewhat fairer.

In the school sports setting, the change centiles offer an objective scale to give children with a poorer starting level, but a clear improvement, feedback that is adjusted to their potential.

Furthermore, the proposed reference centiles offer a diagnostic tool with which to intervene and offer help (e.g., in the case of a severe drop in performance), or as a resource in studying questions relating to “talent-spotting” in competitive in the case of unusually large improvements.

### Strengths and limitations

The DMT is currently used predominantly in German-speaking countries, which is partly due to the fact that the test manual was written in German by the authors of the DMT. However, the individual items of the test can also be found in this or similar form in other test batteries. Regardless of the tests used, it can be stated that when using longitudinal data, greater attention should be paid to the variability of the results.

## Conclusions

The presented tool is based on the presented disciplines (standing long jump, jumping sideways, push-ups, stand and reach, balancing walking backwards) of the DMT. However, the analytical approach adopted here might also be used for other motor test batteries [19]. Thus, this may be a possibility to advance and improve the often requested aspect of harmonisation and calibration of longitudinal approaches [1, 36].

## Supporting information

S1 Table Differentiated no. of participants included. Descriptive statistics for the no. of participants (n) that are included repeatedly in the model.
(TIF)

S2 Table Application example. Realistic example of an evaluation of the changes in performance of a test subject.
(TIF)
