# Supplementary figures and images for: Reference centiles based on year-to-year changes for a longitudinal evaluation of motor performance in children and adolescents
Source: PLoS One. 2022 Jan 7;17(1):e0262163. doi: 10.1371/journal.pone.0262163 (PMC8741030; doi:10.1371/journal.pone.0262163)

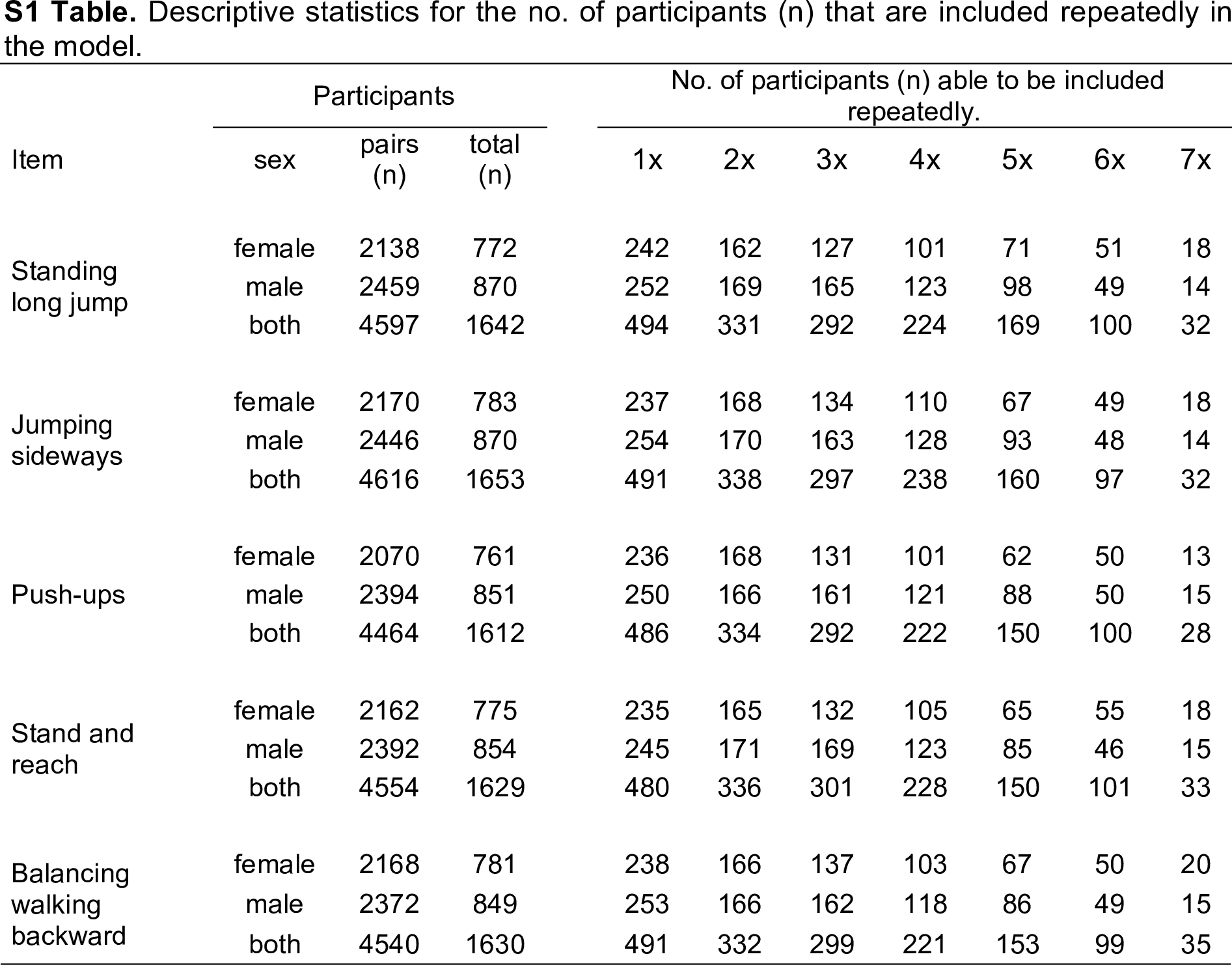

Supplement: S1 Table — Descriptive statistics for the no. of participants (n) that are included repeatedly in the model. (TIF) [file pone.0262163.s001.tif]

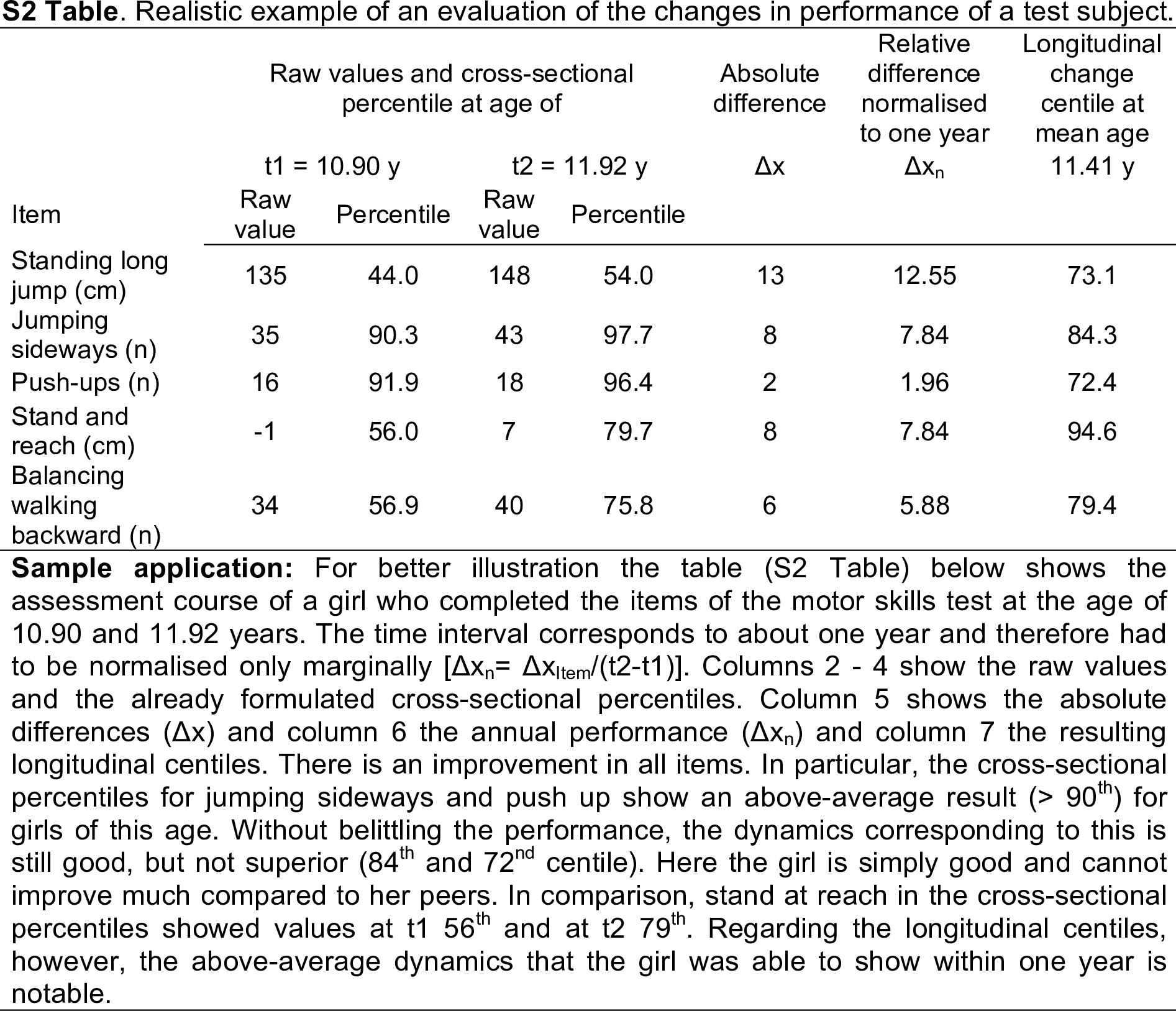

Supplement: S2 Table — Realistic example of an evaluation of the changes in performance of a test subject. (TIF) [file pone.0262163.s002.tif]
